# Label-free distinction of implant infection–associated bacterial biofilms by Mueller matrix polarimetry

**DOI:** 10.1117/1.JBO.30.8.085001

**Published:** 2025-08-22

**Authors:** Gaurav Sharma, Katharina Doll-Nikutta, Hanna Lena Thoms, Maria Leilani Torres-Mapa, Bernhard Roth

**Affiliations:** a Leibniz University Hannover, Hannover Centre for Optical Technologies, Hannover, Germany; b Hannover Medical School, Department of Prosthetic Dentistry and Biomedical Materials Science, Hannover, Germany; c Lower Saxony Center for Biomedical Engineering, Implant Research and Development (NIFE), Hannover, Germany; d Leibniz University Hannover, Institute of Quantum Optics, Hannover, Germany; e Cluster of Excellence PhoenixD, Hannover, Germany

**Keywords:** polarimetry, polarization detection, Mueller matrix, oral bacteria, biofilms

## Abstract

**Significance:**

Bacterial biofilm agglomerates are the cause of hard-to-treat implant-associated infections but currently can only be distinguished using sophisticated microbiological or molecular biological methods. Optical methods can potentially provide a label-free, noncontact approach to detect the presence of bacterial species associated with implant infections that could aid in the early diagnosis of implant-associated diseases.

**Aim:**

Our aim is to measure the polarization signal from implant-associated bacteria biofilms using Mueller matrix polarimetry. Furthermore, we present an analysis of the Mueller matrix element to detect and distinguish the different bacterial biofilm species.

**Approach:**

Several biofilms formed by bacterial species associated with orthopedic (*Staphylococcus aureus* and *Staphylococcus epidermidis*) and dental implants (*Streptococcus oralis, Streptococcus mutans*, and *Porphyromonas gingivalis*) were grown on titanium, a typical implant material. Polarization signals were acquired in a reflection mode using a calibrated polarimetry setup.

**Results:**

The results show that different biofilms could be qualitatively distinguished using the Mueller matrix element analysis. The values derived from bacterial species measurements were distinctly different from those of the bare titanium discs. From the Lu-Chipman decomposition, parameters such as polarizance and diattenuation were calculated for each of the species.

**Conclusions:**

The results provide deeper insight into the interaction of polarized light with bacterial microcolonies. The physiologically growing biofilms form the basis of their polarimetric response signal. Our approach has potential for fast and nondestructive investigation for implant infection detection, potentially *in situ* and *in vivo*.

## 1 Introduction

Identification, classification, and detection of oral pathogenic biofilms by means of polarimetry is an underexplored field of research. It could significantly contribute to the therapy of hard-to-treat implant-associated infections. Osseointegrated implants, such as dental implants or hip and knee prostheses, are common treatment options in modern medicine. However, as they are made of artificial materials and lack their own immune system, they are prone to bacterial colonization and subsequent infection development.^1^ The reported prevalence of peri-implantitis ranges between 0% and 39.7% within 5 years from the insertion of the dental implant.^2^ In endoprosthetics, more than 10% of patients nationwide require revision surgery.^3^ The etiological reasons for such infections are bacterial biofilms, which are complex syntrophic microbial communities consisting of adherent cells encased within a matrix of extracellular polymeric substances.^4^^,^^5^ This structure, as well as altered metabolism and interspecies support networks, makes biofilms inherently intolerant toward antibiotics and renders the treatment of implant-associated infections challenging.^6^

As demographic shifts and rising life expectancy continue, the demand for biomedical products is projected to grow steadily in the coming years. A significant portion of this demand is attributed to dental materials, with dental prostheses comprising nearly 50% of the total count. Currently, over 1.3 million dental implants as well as more than 400,000 hip and knee joints are placed annually in Europe, and this figure is anticipated to rise further.^3^^,^^7^^,^^8^ Titanium and its alloys are the primary biomedical material for such implants because of good bio-compatibility, mechanical properties, and good corrosion resistance. However, this material has no antibacterial properties, and surface modifications would be necessary to inhibit microbial accumulation. Therefore, early detection of bacterial surface colonization is still a central requirement for successful infection therapy.^9^^,^^10^ In line with this, identification of the respective colonizing species is of significant importance.

For endoprostheses, commensal *Staphylococcus* species are the major cause of infection.^11^ By contrast, dental implants face the highly complex oral microbiome that, in addition, changes in its composition with infection progression. Common early colonizers are *Streptococcus* species, whereas oral pathogens such as *Porphyromonas gingivalis* appear at later stages.^12^ Their current identification requires more elaborate microbiological or molecular biological techniques.^13^

Promising bacterial detection methods that offer high efficiency^14^ and accuracy without requiring extensive sample preparation, complex measurement procedures, or specialized equipment would be highly valued as they have the potential to advance current knowledge and expand the boundaries of the field. Optical methods are extensively utilized for the analysis of biofilms, offering noninvasive and real-time insights into their structure, composition, and dynamics. These techniques are pivotal in understanding biofilm behavior across various environments, including medical, industrial, and environmental settings.^15^^–^^17^ Clinical mapping of oral biofilm has been primarily restricted to macroscopic plaque staining techniques and bacterial biofluorescence. Among the most commonly used microscopy techniques are confocal laser scanning microscopy and fluorescence *in situ* hybridization. The former gives a three-dimensional visualization of biofilms, and the latter combines fluorescence microscopy with nucleic acid probes to identify and localize specific microbial populations within biofilms.^16^ Flow cells are best suited for continuous and *in situ* microscopic biofilm monitoring as they can easily be integrated into systems.^18^ Biomolecular staining used to enhance the visibility of the cells, nucleic acids, and proteins that make up biofilms, followed by comparing optical images, has been used for rapid quantification of biofilm growth (measurement of cell density).^19^ Several oral planktonic bacterial species were detected using a functionalized gold nanoparticle biosensor array due to the differences in the aggregation of the nanoparticles on the bacteria’s surface, which led to a unique plasmonic shift.^20^ Raman spectroscopy enables identification and analysis of both planktonic and biofilm chemical and metabolic patterns, as well as biofilm activity. Different bacterial phenotypes have been identified, providing comprehensive datasets using Raman spectroscopy combined with confocal microscopy. However, Raman spectroscopy tends to have a low signal-to-noise ratio, background fluorescence, and a long measuring time.^21^

Mueller matrix polarimetry (MMP) has been used as a label-free tool for biomedical diagnosis because of its sensitivity to microstructural changes in tissues.^22^^–^^24^ Applications of Mueller matrix (MM) analysis include early detection of cancer, assessment of burn injuries, and evaluation of fibrotic tissue, where the polarization signatures provide critical diagnostic information.^25^^–^^28^ In biomedical optics, MM imaging has been widely utilized to assess tissue birefringence, scattering, and depolarization effects, enabling noninvasive evaluation of structural and pathological changes in biological samples.^29^^,^^30^ Compared with other optical techniques, the Mueller matrix offers a more detailed characterization of tissue by capturing both amplitude and polarization-related changes, making it a versatile approach for understanding complex biological interactions.^31^^,^^32^ In the field of bacterial biofilms, MMP has been employed to detect and discriminate bacterial colonies grown on agar medium.^33^ The study primarily relied on imaging data derived from Mueller matrix analysis and polar decomposition, complemented by frequency distribution histograms. In another investigation, polarimetry techniques were used to analyze scattering patterns of a single bacterial species for detection purposes.^34^ Adaptation of MM to an endoscope would bridge the clinical translation of the approach *in vivo*. Polarimetric endoscopes have been developed for tissue characterization, offering both *in vivo* and *ex vivo* capabilities. In addition, endoscopes can be tailored to measure areas of 3×3 or 4×4 MM. Besides having imaging capabilities, they are suited to measure linear and circular polarization, directional birefringence, and dichroism.^35^^,^^36^ Similar polarimetric approaches have been employed to characterize polycrystalline biological films derived from dried plasma, tear films, peritoneal fluid, and urine. These systems exhibit complex optical anisotropy combining linear/circular birefringence and dichroism, which has been modeled through rotation-invariant MM elements and quantified using statistical moments. Such approaches have successfully differentiated healthy donors from patients with conditions, such as endometriosis or albuminuria, based on microstructural differences in film morphology and optical activity.^37^^,^^38^

To date, the application of MMP analysis in the context of bacteria growing in infection-relevant biofilm morphologies has not been reported. The present study was undertaken with the objective of exploring the potential of MMP for the label-free identification of different implant infection–associated bacterial biofilms.^39^ Hence, the following bacterial species were selected for the study, *Staphylococcus aureus (S. aureus), Staphylococcus epidermidis (S. epidermidis), Porphyromonas gingivalis (P. gingivalis), Streptococcus mutans (S. mutans)*, and *Streptococcus oralis (S. oralis)*, and grown on the implant material titanium. They are abbreviated as *Sa*, *Se*, *Pg*, *Sm*, and *So* in the figures, respectively. The samples were characterized by confocal microscopy for biofilm volume and bacterial viability. Bacterial samples, in conjunction with blank titanium as the control sample (abbreviated as Ct), were probed by polarimetry. The unique polarization responses of the biofilms were based on the interplay of the microstructures and the bacterial colonies’ growth. Furthermore, statistical analysis was performed to determine if the differences between each species and the control samples were statistically significant. Our work demonstrates that MMP can be a useful label-free method in detecting the presence of implant-associated biofilms on implant materials and also to differentiate among different species.

## 2 Materials and Methods

### 2.1 Bacterial Strains and Culture Conditions

The used bacterial strains and culture conditions are given in Table 1. In summary, the strains were stored as glycerol stocks and pre-cultivated for every experiment. Bacteria were harvested by centrifugation and adjusted for optical density at an optical wavelength of 600 nm $\left({\mathrm{OD}}_{600}\right)$ in their respective biofilm medium. Biofilms were formed on 12 mm titanium grade IV discs ($R_{a}=0.3\pm 0.05\;\mu m$). To prepare an additional monolayer of *S. epidermidis*, bacteria were pre-cultured, as described in Table 1, adjusted to an ${\mathrm{OD}}_{600}$ 0.05 in phosphate-buffered saline and incubated statically for 3 h at 37°C under aerobic conditions. Notably, *S. aureus* biofilms have a lower starting ${\mathrm{OD}}_{600}$ of 0.001 compared with the other species (${\mathrm{OD}}_{600}$ of 0.05), which may contribute to differences in biofilm volume and thickness.

**Table 1:** Bacterial strains and biofilm culture conditions.

Bacterial strain	Abbreviation	Pre-culture medium	Pre-culture conditions	Biofilm medium	Biofilm ${\mathrm{OD}}_{600}$	Biofilm conditions
*Staphylococcusaureus* DSM 799^a^	*Sa*	TSBy^c^	16 h aerobe shaking at 37°C	TSBy^c^ + 50 mM glucose	0.001	24 h aerobe static at 37°C
*Staphylococcusepidermidis* DSM 20044^a^	*Se*	TSBy^c^	16 h aerobe shaking at 37°C	TSBy^c^ + 50 mM glucose	0.05	24 h aerobe static at 37°C
*Streptococcusoralis* ATCC 9811^b^	*So*	THBy^d^	18 h microaerophile shaking at 37°C	TSBy^c^ + 50 mM glucose	0.05	48 h anaerobe static at 37°C
*Streptococcusmutans* DSM 20523^a^	*Sm*	THBy^d^	18 h microaerophile shaking at 37°C	THBy^d^ + 5% Saccharose	0.05	48 h anaerobe static at 37°C
*Porphyromonasgingivalis* DSM 20709^a^	*Pg*	FAB^e^	24 h anaerobe static at 37°C	BHI^f^ + 10 μg/mL vitamin K	0.05	48 h anaerobe static at 37°C

a German Collection of Microorganisms and Cell Cultures GmbH, Braunschweig, Germany.

b American Type Culture Collection, Manassas, Virginia, United States.

c Tryptic Soy Broth (Oxoid Limited, Hampshire, United Kingdom) supplemented with 10% yeast extract (Carl Roth GmBH & Co. KG, Karlsruhe, Germany).

d Todd Hewitt Broth (Oxoid Limited) supplemented with 10% yeast extract.

e Fastidious Anaerobe Broth (Oxoid Limited).

f Brain Heart Infusion (Oxoid Limited).

Biofilms were stained using the LIVE/DEAD BacLight bacterial viability kit (Life Technologies, Darmstadt, Germany) according to the manufacturer’s instructions, applying both dyes in a 1:2000 dilution. Afterward, biofilms were fixed in 2.5% glutardialdehyde and covered with phosphate-buffered saline for microscopy. Images were taken using a confocal laser scanning microscope (TCS SP8, Leica Microsystems, Mannheim, Germany) with 488 nm and 552 nm laser lines for excitation and emission detection at 500 to 550 nm and 650 to 750 nm. From each sample (N=4), three images were taken ($N_{\text{Total}}=12$) with an image size of $295\times 295\;{\mu m}^{2}$ and a z-step-size of 5  μm. From these images, the biofilm volume and bacterial viability were calculated using the Imaris software package v8.4.1 (Bitplane AG, Zurich, Switzerland). Results were statistically compared using a one-way ANOVA test. Figures 2(b) and 2(c) show the biofilm volume and live–dead distribution for each species, together with ANOVA p-value heat maps. In addition, representative images of *S. epidermidis* adhesion were taken with an image size of $190\times 190\;{\mu m}^{2}$. Most biofilms consist of ∼10% bacterial cells and 90% extracellular matrix by dry mass.^40^ *S. aureus* and *S. epidermidis* are spherical in shape, with diameters ranging from 0.5 to 1  μm. *S. oralis* is similarly spherical and falls within the same size range. *S. mutans* is typically spherical, measuring 0.5 to 0.75  μm in diameter, though under certain conditions it can exhibit a short rod-like shape, with lengths up to 1  μm. *P. gingivalis* is rod-shaped, with a typical width of 0.5  μm and a length ranging from 1 to 3  μm.^41^ As the bacteria are all very similar in shape and size, except for *P. gingivalis*, an effect based purely on that is unlikely. The average biofilm thickness, based on z-stack confocal imaging, ranged from 80 to 100  μm. The scattering regime in a biological sample depends on the size parameter. For particles much smaller than the wavelength, Rayleigh scattering dominates, producing symmetric angular scattering and strong wavelength dependence. By contrast, Mie scattering occurs when the particle size is comparable to or larger than the wavelength, typical for bacterial cells (0.5 to 2  μm) in the visible wavelength range. Mie scattering is strongly forward-directed, weakly wavelength-dependent, and often leads to significant polarization, especially in optically dense and structurally heterogeneous media such as biofilms.^27^ The structural depth and the dense extracellular matrix (ECM) composition of our samples suggest that both single and multiple scattering contribute to the observed Mueller matrix response, with multiple scattering likely dominating in the thicker biofilms.

### 2.2 Mueller Matrix Polarimetry

**Fig. 1:**
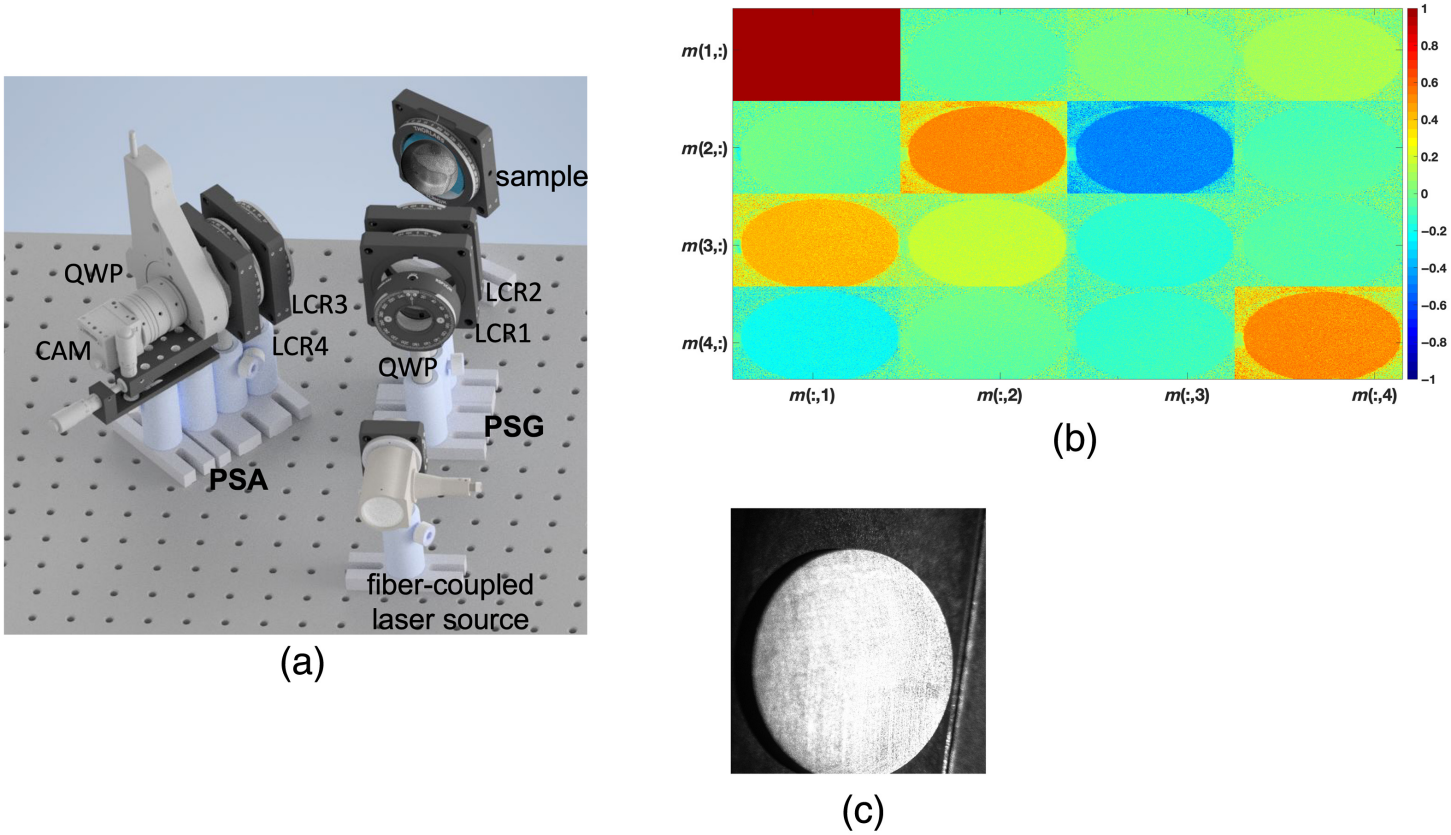
(a) Scheme of Mueller matrix polarimetry setup containing different polarizers, to acquire either 16 or 36 images from the camera. PSG is the polarization state generator consisting of quarter-wave plate and two liquid crystal retarders (LCR 1, 2). PSA is the polarization state analyzer with LCR 3, 4, which is the same as the PSG and has a CMOS camera (CAM) for imaging. (b) MM of the above sample, with color bar. (c) A representative image of a titanium disc with grown *Streptococcus oralis*.

An MM imaging polarimeter is utilized to capture the spatially varying polarization properties of optical systems. In this study, we employed an experimental setup similar to the one described in our previous works.^42^^,^^43^ The wide-field imaging setup consists of two main arms: the polarization state generator (PSG) arm and the polarization state analyzer (PSA) arm [Fig. 1(a)].

The PSG arm is responsible for illuminating the material under investigation with a series of pre-determined polarization states. The sample is illuminated using a continuous wave laser fiber-coupled light with a wavelength of 632 nm and a power of 0.5 mW. PSG comprises a quarter-wave polarizer and two liquid crystal retarders, which collectively generate six specific polarization states. The optical signal reflected by the material under investigation is collected through the PSA arm. The optical elements in the PSA arm are identical to those in PSG, with the exception of an additional CMOS imaging camera for signal acquisition. For each sample, a total of 36 images were acquired, with the acquisition time being less than 1 s. The measurement angle between the PSG and PSA arm is maintained at 20 deg. Within this range, the polarization angles are suitable for performing post-processing of the data. Angles that are too narrow or too wide can result in grazing incidence or emission of polarized light on the sample, thereby affecting the measurement results. A photograph of the titanium disc with grown biofilm is shown in Fig. 1(c). From the acquired polarization signals, we calculate the MM elements values, which range from −1 to 1. We calculate the MM elements’ value with mathematical relations as specified in previous work.^42^ An example of the MM imaging is shown in Fig. 1(b) for *S. oralis* grown on a titanium disc.

The optical setup was calibrated to ensure the reliability and reproducibility of the results. The calibration relies on applying suitable values for the supply voltages of the liquid crystal retarders (LCRs) to achieve $\frac{\lambda }{4}$ and $\frac{\lambda }{2}$ phase delays, respectively. All angles are aligned with respect to the first polarizer in the PSG, which has a fixed orientation during the whole process. The second polarizer is mounted in a motorized rotating holder and is then oriented perpendicular to the first one. The first polarizer was adjusted to 45 deg manually, after which we performed the following calibration sequence: (1) calibration of the fast axes of all LCRs to 0 deg; (2) determination of $\frac{\lambda }{2}$ phase retardation for all LCRs through analysis of the voltage–intensity relationship at the sensor; and (3) configuration of a $\frac{\lambda }{4}$ phase retardation for LCR2 and LCR3 utilizing a quarter-wave-plate. Throughout this process, the imaging camera served as the measurement sensor for intensity readings.

In Mueller matrix polarimetry, the interaction between the incident polarization state and the sample is described by a 4 4 real matrix known as the MM. For a more comprehensive understanding of Mueller matrix polarimetry, detailed discussions can be found in the literature.^44^

When an incoming light is represented by a 4×1 Stokes vector (S), the resulting polarization state transformation is given by $S^{\prime }=MS$, where $S^{\prime }$ is the outgoing Stokes vector and M is the Mueller matrix as

$$(\begin{array}{c} S_{o0}\\ S_{o1}\\ S_{o2}\\ S_{o3} \end{array})=[\begin{array}{cccc} m11 & m21 & m13 & m14\\ m21 & m22 & m23 & m24\\ m31 & m32 & m33 & m34\\ m41 & m42 & m43 & m44 \end{array}](\begin{array}{c} S_{i0}\\ S_{i1}\\ S_{i2}\\ S_{i3} \end{array}).$$ (1)

The MM contains all the essential information on how the sample alters the polarization state of the incident light. However, the MM elements obtained from the experiments do not provide information about the sample’s physical properties. Therefore, it is necessary to decompose the MM into its various known contributions, as is possible with the polar decomposition method proposed by Chipman, as follows:^45^

$$M_{\exp}=M_{\Delta }\cdot M_{R}\cdot M_{D}.$$ (2)

$M_{\exp}$ is the experimentally measured MM, $M_{D}$ is the diattenuation matrix, $M_{R}$ is the retardance matrix, and $M_{\Delta }$ is the depolarization matrix. The depolarization (Δ) and the decomposition parameters, diattenuation (D), the total retardance (R), and the polarizance (P), can be calculated as follows:

$$D=\frac{1}{m11}\sqrt{m12^{2}+m31^{2}+m41^{2}},$$ (3)

$$\Delta =1-\frac{\left|tr(M_{\Delta })-1\right|}{3},$$ (4)

$$R=\cos^{-1}(\frac{tr(M_{R})}{2}-1),$$ (5)

where tr(M) is the trace of the matrix M,

$$P=\frac{1}{m11}\sqrt{m21^{2}+m31^{2}+m41^{2}}.$$ (6)

Although diattenuation describes the difference in attenuation between orthogonal and polarization states, retardation refers to the difference in phase accumulation between two polarization states, and polarizance refers to the change of completely unpolarized light to polarized light. In addition, depolarization quantifies the fraction of light lost or transformed into unpolarized light. In addition, for a nondepolarizing medium, the total retardance matrix (both linear and circular) $M_{R}$ is written as

$$M=M_{R}M_{D},$$ (7)

$$M_{R}=MM_{D}^{-1},$$ (8)

$$M_{R}=[\begin{array}{cc} 1 & {\overset{\to }{0}}^{T}\\ \overset{\to }{0} & m_{R} \end{array}].$$ (9)

$m_{R}$ is a matrix obtained from the lower 3×3 part of the MM. An intuitive understanding is to know that retardance is a function of the lower 3×3 elements of the MM.

## 3 Results and Discussion

### 3.1 Biofilm Characterization

**Fig. 2:**
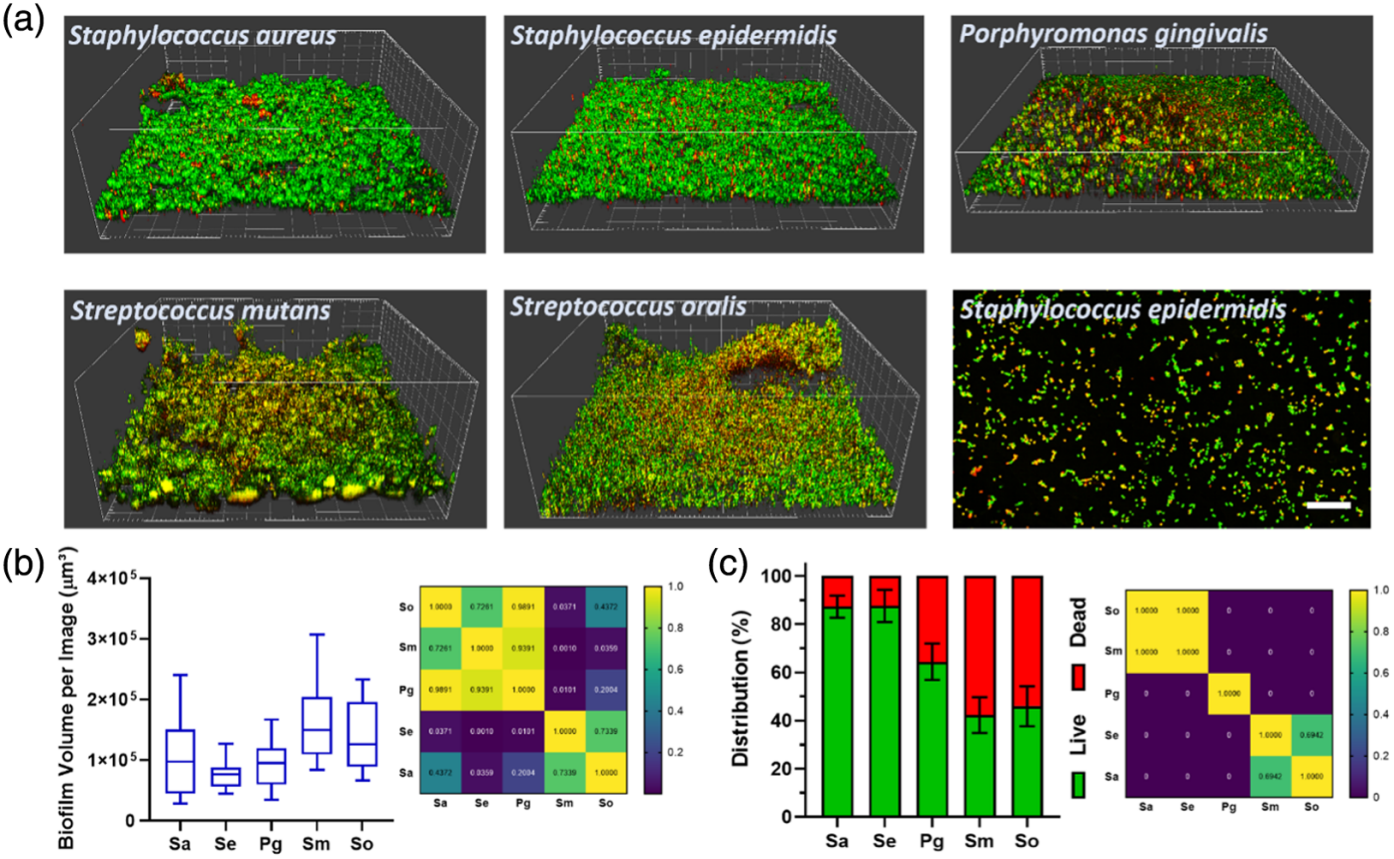
Biofilm characterization. (a) Representative 3D reconstructions of biofilms of the respective species, as well as adhesion of *S. epidermidis*. Viable bacteria are depicted in green; impaired bacteria are depicted in yellow/red. Biofilm box length corresponds to 295  μm. Adhesion scale bar corresponds to 20  μm. (b) Boxplots of biofilm volume and p-value heat maps after one-way ANOVA test (right panel). (c) Mean ± standard deviation of viability distribution of the different species’ biofilms and p-value heat maps after one-way ANOVA test (right panel). The blue scale denotes a statistically significant difference.

The aim of the present study was to establish whether Mueller matrix polarimetry can distinguish among different implant infection–associated bacterial biofilms. These biofilms were grown on implant-grade titanium disks and were initially characterized by viability, fluorescence staining, and confocal microscopy. As shown in Fig. 2(a), all biofilms exhibited a multilayered, three-dimensional morphology, which is typical for mature biofilms.^46^ However, the exact topography of the biofilms differed depending on the bacterial species, where *S. epidermidis* and *P. gingivalis* showed flat, more compact topographies, *S. aureus* and *S. mutans* biofilms formed protruding clusters, and *S. oralis* biofilms exhibited a generally loosened structure. These differences in topography are in line with the calculated biofilm volume, which was significantly (p≤0.05) larger for the more structured biofilms [Fig. 2(b)]. Differences in biofilm architecture have already been shown for several oral bacteria grown in a flow chamber and are associated with species-specific differences in biofilm matrix macromolecules.^46^^,^^47^

Cell viability is a factor that influences the polarization response. The LIVE/DEAD BacLight bacterial viability assay was used to distinguish between intact (viable) and membrane-compromised (nonviable) bacteria within the biofilms. The viable (LIVE) bacterial cells generally maintain structural integrity, preserving their characteristic size, shape, and refractive index distribution, all of which influence scattering behavior.^48^^,^^49^ Dead cells exhibit different morphologies, potentially modifying the anisotropy and scattering cross-section. Such changes can influence the polarization behavior by either increasing random scattering from fragmented material or reducing anisotropic signatures from organized colonies. Smaller fragments resulting from lysis may shift the dominant scattering regime toward Rayleigh-like behavior, characterized by more symmetric and less polarization-altering scattering, whereas intact live cells continue to scatter via the Mie regime, contributing to strong forward scattering and depolarization.^27^^,^^50^ The fixation of samples preserves the structural and morphological features while halting metabolic activity and preventing further cell lysis or degradation.

Bacterial viability displayed a genus-specific pattern, with significantly (p≤0.05) decreasing viability from *Staphylococci* to *P. gingivalis* to *Streptococci* [Fig. 2(c)]. This pattern can be linked to the natural growth conditions of these bacteria, with *Staphylococci* typically forming monospecies biofilms^11^ and oral bacteria, such as *P. gingivalis* and *Streptococci*, growing in multispecies biofilms, where metabolic interactions among species significantly enhance biofilm viability.^51^ It would be noteworthy to mention cell viability as a factor that alters the morphology and scattering cross-section of the cell structures [see Fig. 2(c)]. We note that *Streptococci* with higher nonviable count (DEAD) show more distinct MM values than the *Staphylococci*, which have higher viable cell count (LIVE).

The ECM is known to vary across species and likely contributes to the observed differences in polarimetric response. *S. mutans* and *S. Oralis* produce a dense polysaccharide-rich matrix, whereas *S. epidermidis* and *S. aureus* form ECMs with more proteinaceous content.^52^^,^^53^ *P. gingivalis* biofilms include extracellular vesicles and proteolytic components.^54^ These compositional differences can influence volumetric scattering and birefringence through variations in refractive index and internal structure, even if not directly visualized in this study.^51^

### 3.2 Multilevel Species Comparison by Polarimetry

**Fig. 3:**
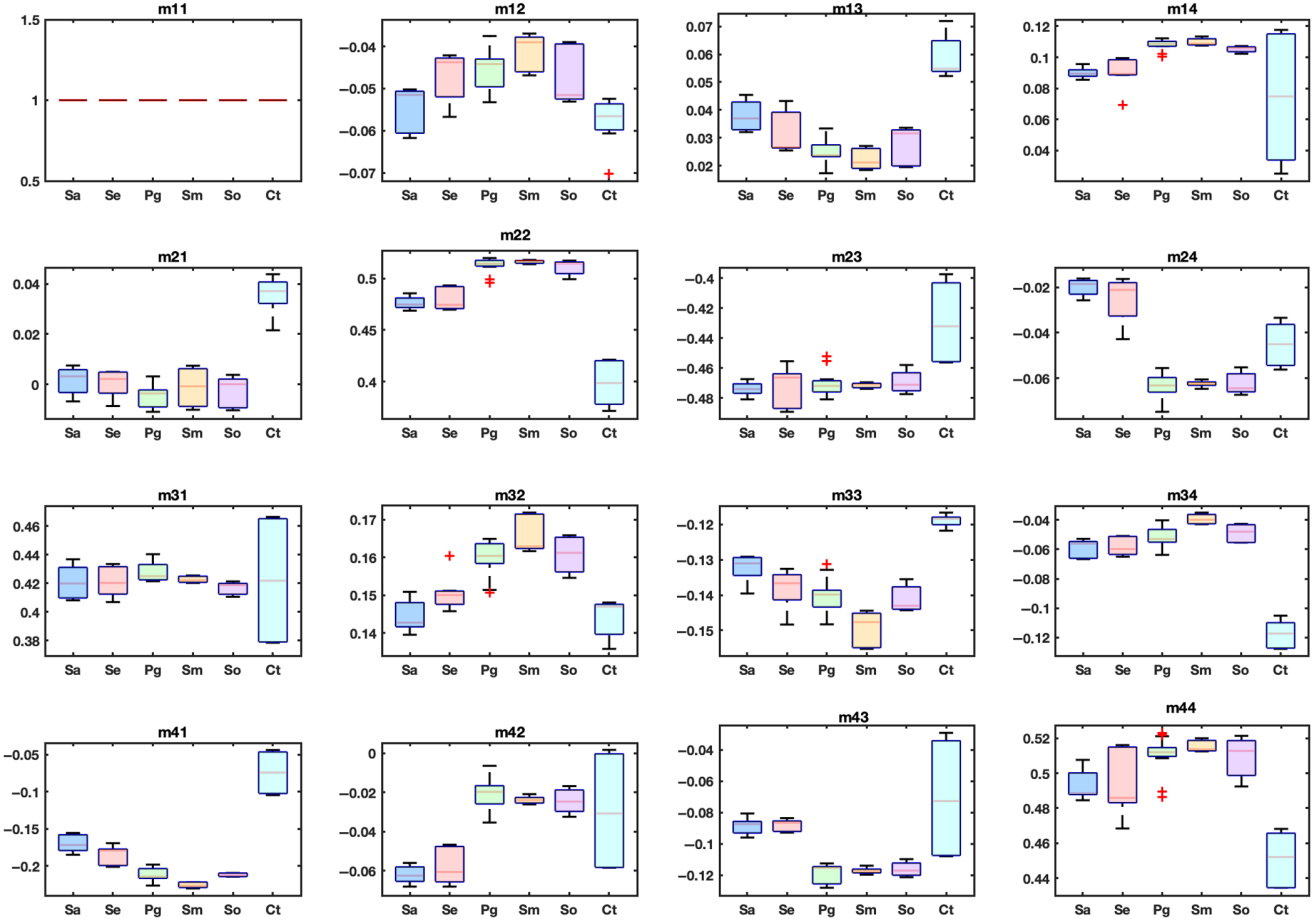
Comparison of the MM values of the bacterial species *Staphylococcus aureus*, *Staphylococcus epidermidis*, *Porphyromonas gingivalis*, *Streptococcus mutans*, *Streptococcus oralis* (*Sa*, *Se*, *Pg*, *Sm*, *So*), and the control sample (Ct). Each bacterial species has a unique polarization response that is distinctly different from the Ct sample. The box plots are measurements for three titanium disks per species, with four to five measurements performed on each sample.

The described biofilms, grown on titanium discs, as well as the blank titanium discs used as a control, were probed by MMP. The polarization responses of the bacterial biofilms and the control were analyzed by combined box plots of the MM values, as shown in Fig. 3. In the species versus control comparison, the MM values of the control group did not match those of the species. The control group was clearly distinguishable from the rest of the species for the diagonal elements (m22, m33, and m44). In Fig. 3, we note that for m22, all of the species values lie within the range between 0.45 and 0.50, whereas the Ct values are below 0.40. This indicates that Ct is differentiated from the bacterial species. With the exception of some elements, all nondiagonal elements exhibited this differentiation within the error. The values for the elements m13, m14, m21, m23, m32, m34, m41, and m43 of the species are outside the range of values measured for the control group. The values exhibited by the elements m12, m24, m31, and m42 of the bacterial biofilms were similar to the values exhibited by control sample, although distinction too was noted. Thus, the differentiation here is not as pronounced. This measurement thus served to reinforce the conclusion that the polarization signal was derived from the bacterial films. The titanium substrate had a uniform roughness, which may contribute to baseline values of the control samples. However, the distinctly different MM signatures observed in biofilms suggest that the polarization signal primarily arises from the biofilm’s volumetric composition, including its thickness, density, and internal structure, rather than from surface topology alone.

The bacterial species *Streptococcus oralis*, *Streptococcus mutans*, and *Porphyromonas gingivalis* had among the lowest mean values for m33 of all the species for m33 and among the highest for m22 and m44. We observed that *Staphylococcus aureus* and *Streptococcus oralis* have very similar mean values for the diagonal elements. For comparison among the bacterial species, the MM values exhibited comparable characteristics with substantial differentiation among samples. It was noteworthy that all MM values were unique within the experimental error limits, thereby indicating distinct polarization responses. The high diagonal element values suggest a strong birefringence behavior or low depolarization power. Unequal diagonal element values indicate that the bacterial colonies exhibited different depolarizing capabilities. For isotropic samples, nondiagonal values are typically low or approaching 0, and m22 and m33 are approximately equal. The presented data strongly support an anisotropic behavior, evidenced by the nonzero and nondiagonal element values and high diagonal element magnitudes.

Many physical factors are at play, affecting the polarization response of the bacterial films. These include the thickness or the density of the biofilm, the size of the bacterial colonies, the adhesion of the bacteria to the titanium discs, and the growth of bacterial colonies. The growth rate of the bacteria contributes heavily to the size of the colony and its density. It has been demonstrated that bacteria with low density form large colonies and vice versa.^55^ However, it must be noted that biofilms grown on the titanium discs may exhibit a varying polarization response compared with biofilms grown on agar medium. The species in our study differ not only in ECM composition but also in bacterial cell shape and size, which influence their optical scattering properties. The high ECM content in biofilms (typically 90% of dry mass) contributes to internal refractive index heterogeneity, enhancing volumetric scattering and depolarization. These structural differences provide a physical basis for the species-specific MM responses observed in our data, including variations in diagonal and off-diagonal elements as well as decomposition parameters.

**Fig. 4:**
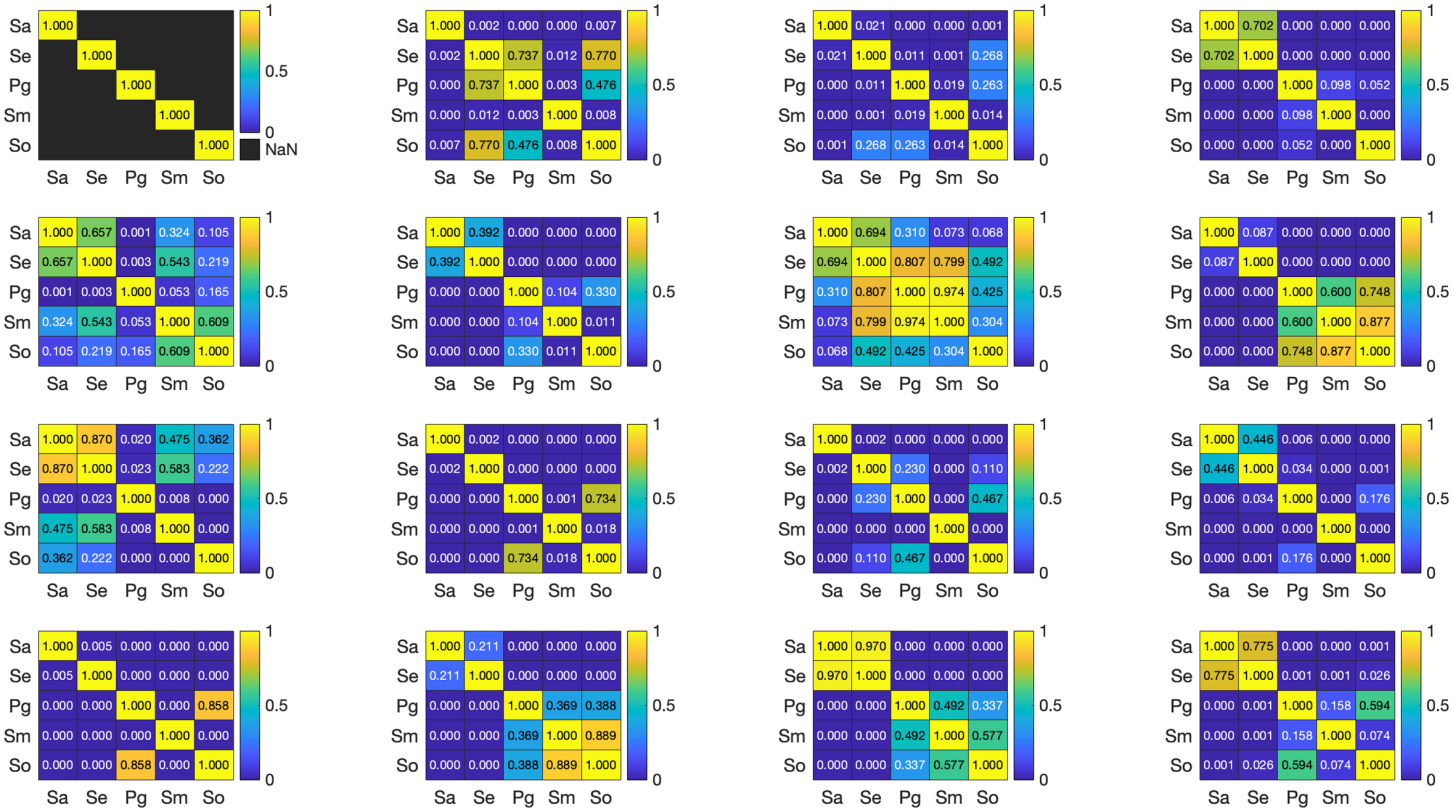
ANOVA results of the *Sa*, *Se*, *Pg*, *Sm*, and *So* bacterial biofilm (*Staphylococcus aureus*, *Staphylococcus epidermidis*, *Porphyromonas gingivalis*, *Streptococcus mutans*, *Streptococcus oralis*, respectively). The blue scale indicates larger statistically significant differences (low p-value), and the yellow scale indicates less statistically significant differences (high p-value).

A thorough quantitative analysis would help provide deeper insights into the nature of the biofilm’s polarization response. A one-way ANOVA test was conducted to compare the mean polarization responses across five bacterial species and the control group to determine whether significant differences exist among the groups. The dataset was collected from three titanium discs per species, with 4 to 5 measurements taken per disc, resulting in ∼12 to 15 measurements per species (n∼15). To visualize the variation in polarization responses among the bacterial species and the control, heat maps of individual MM elements were generated, as shown in Fig. 4. These visualizations were complemented by statistical analysis using one-way ANOVA to determine the significance of observed differences. The control group consistently exhibited higher values compared with the bacterial species for all three diagonal elements. The differences were most pronounced for m22, where the control group showed mean differences exceeding 0.1 compared with several bacterial species, followed by m33 and m44. This suggests a fundamental difference in optical properties or surface interactions between the control and bacterial samples (see Fig. 3). For clearer presentation and explanation of the oral bacterial film species’ polarization response, we have excluded the control disc from Fig. 4 and from the heat maps. Figure 4 shows the p-values of the mean MM values for each of the species. The darker blue colors on the scale represent lower p-values, indicating more significant differences, whereas the light yellow colors represent higher p-values, indicating that the bacterial species are not significantly different. The F-value is a test statistic for ANOVA to determine the significance of the model or a component of the model. A higher F-value suggests more significant differences among groups relative to within-group variation. *Post hoc* tests provide confidence intervals and the mean differences among species. If the confidence interval for a pair does not include 0, it indicates a significant difference. For the diagonal elements m22, m33, and m44 (p<0.05), these elements exhibit the highest mean values among the species, with F-values of 207, 66, and 47, respectively, demonstrating a strong and unique response to polarized light. *Post hoc* analysis using Tukey’s honestly significant difference further identified significant pairwise differences among several groups. The Supplementary Material to this work includes the element-wise mean differences of the MM elements across all species from the *post hoc* analysis, along with the corresponding F-values.

Birefringence is a differential phase shift among orthogonal polarization components as light propagates through an anisotropic medium. It can arise due to structural anisotropy, molecular orientation, or stress within biological samples. A slightly simplified but insightful way to understand the birefringent property is to focus on the off-diagonal elements.^56^^,^^57^ For a homogeneous medium, linear birefringence introduces nonzero values in off-diagonal elements such as m34, m43, m24, and m42, which represent the coupling between circular and ±45  deg linear polarization states, as well as elements within the retardance submatrix. In our study, nonzero off-diagonal values were observed, indicating birefringent behavior of the biofilms. In isotropic chiral media, both effects may co-occur, but in scattering or anisotropic biological media such as bacterial films, apparent circular signatures can also arise due to multiple scattering or the convolution of linear retardance with depolarization.^58^

For m33, the significantly higher m32 compared with m33 may indicate anisotropic scattering or birefringence effects within the sample. This finding suggests that the internal structure or alignment of the bacterial biofilm preferentially influenced the propagation and polarization of circularly polarized light. A comparison of the conjugate pairs, m24 and m42, as well as m34 and m43, provided insights about the birefringence properties of the oral biofilms. The bacterial biofilms’ mean values of these elements were in a similar range, confirming that they have strong birefringent behavior. Further ascertaining the birefringent responses, *Staphylococcus aureus* and *Staphylococcus epidermidis* had values that are significantly different from the values of *Streptococcus oralis*, *Streptococcus mutans*, and *Porphyromonas gingivalis*. Thus, the former two species can be distinguished from the latter species. *Staphylococcus aureus* and *Staphylococcus epidermidis* have very similar birefringent responses owing to their similar shape and colony growth structures on the discs. The remaining species, *Streptococcus oralis*, *Streptococcus mutans*, and *Porphyromonas gingivalis*, had higher p-values (in the yellow region, see Fig. 4), indicating unique MM values or polarization responses of the species. Such a similarity in polarization signal response points to a similar birefringent response. It would be noteworthy to mention cell viability as a factor that alters the morphology and scattering cross-section of the cell structures [see Fig. 2(c)]. We note that *Streptococci* with higher nonviable count (DEAD) show distinct MM values than the *Staphylococci*, which have higher viable cell count (LIVE).

**Fig. 5:**
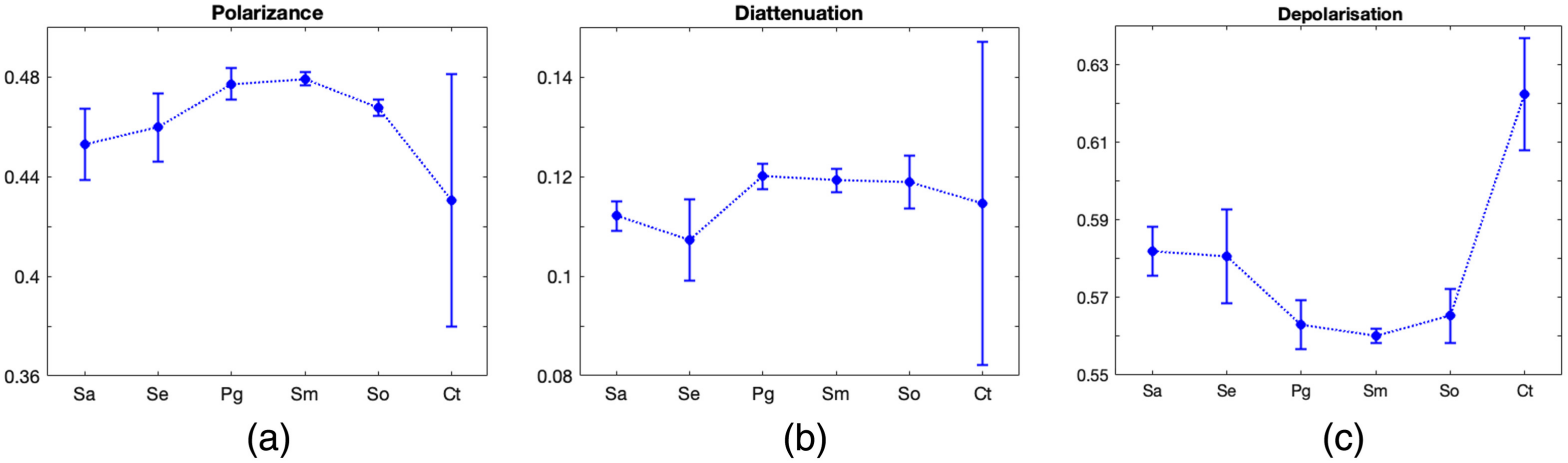
Comparison of the decomposition parameters: (a) polarizance, (b) diattenuation, and (c) depolarization values of the oral bacterial biofilms, *Staphylococcus aureus* (*Sa*), *Staphylococcus epidermidis* (*Se*), *Porphyromonas gingivalis* (*Pg*), *Streptococcus mutans* (*Sm*), *Streptococcus oralis* (*So*), and the control sample (Ct).

The values of m23 demonstrate negligible variation among species; however, for m42, m43, and m44, the grouping of values across species, as previously discussed, is evident. The retardance parameter, derived from the off-diagonal elements of the lower-right submatrix of the MM [the lower right 3×3 matrix in the Mueller matrix, see Eq. (9)], was computed for all samples. However, its values did not exhibit meaningful variation across the bacterial species, and no consistent trend could be identified. As such, it was excluded from further comparative analysis. Referring to Figs. 5(a) and 5(b), the values for polarizance and diattenuation for bacterial species are unique and clearly separated from Ct. The polarization values, although being low in comparison with Ct, show the same unique response for each species [Fig. 5(c)]. The polarization depends on the size of the bacteria and the density of the colony. A higher density of bacteria would lead to lower polarization. Figure 5 indicates that the Ct sample lacks bacterial colonies, whereas the other samples exhibit bacterial growth. In our study, a decrease in polarization was observed when comparing the control surface to those covered with emerging bacterial biofilms. This trend likely reflects a structural transition from a relatively disordered and isotropic surface to one dominated by increasingly organized bacterial microcolonies. Such an organization suppresses random multiple scattering and introduces local anisotropy, leading to more coherent polarization interactions. However, this trend does not persist for larger growth. The higher densities or colony agglomeration can suppress the scattering signals, possibly due to multiple scattering events. This is further supported by studies on bacterial biofilms and colonies, where morphological and structural changes influence polarization parameters, such as linear polarization.^33^ In nutrient-rich environments, bacteria grow in a disordered, sparse manner, resulting in low diattenuation and a relatively high depolarization due to randomized scattering and isotropic index fluctuations. In glucose-enriched media, bacteria exhibited cooperative raft-like arrangements, which increased birefringence and diattenuation due to the emergence of anisotropic domains, whereas the denser cell packing enhanced depolarization.^34^ The study took into account the control sample’s roughness and its polarimetric response. A nonbiological corollary can be found in granular anisotropic films, where variations in thickness and density have been shown to affect depolarization.^59^ Similarly, in biological tissues, microstructural features directly impact polarization and depolarization properties.^60^

### 3.3 Monolayer and Biofilm Comparison

**Fig. 6:**
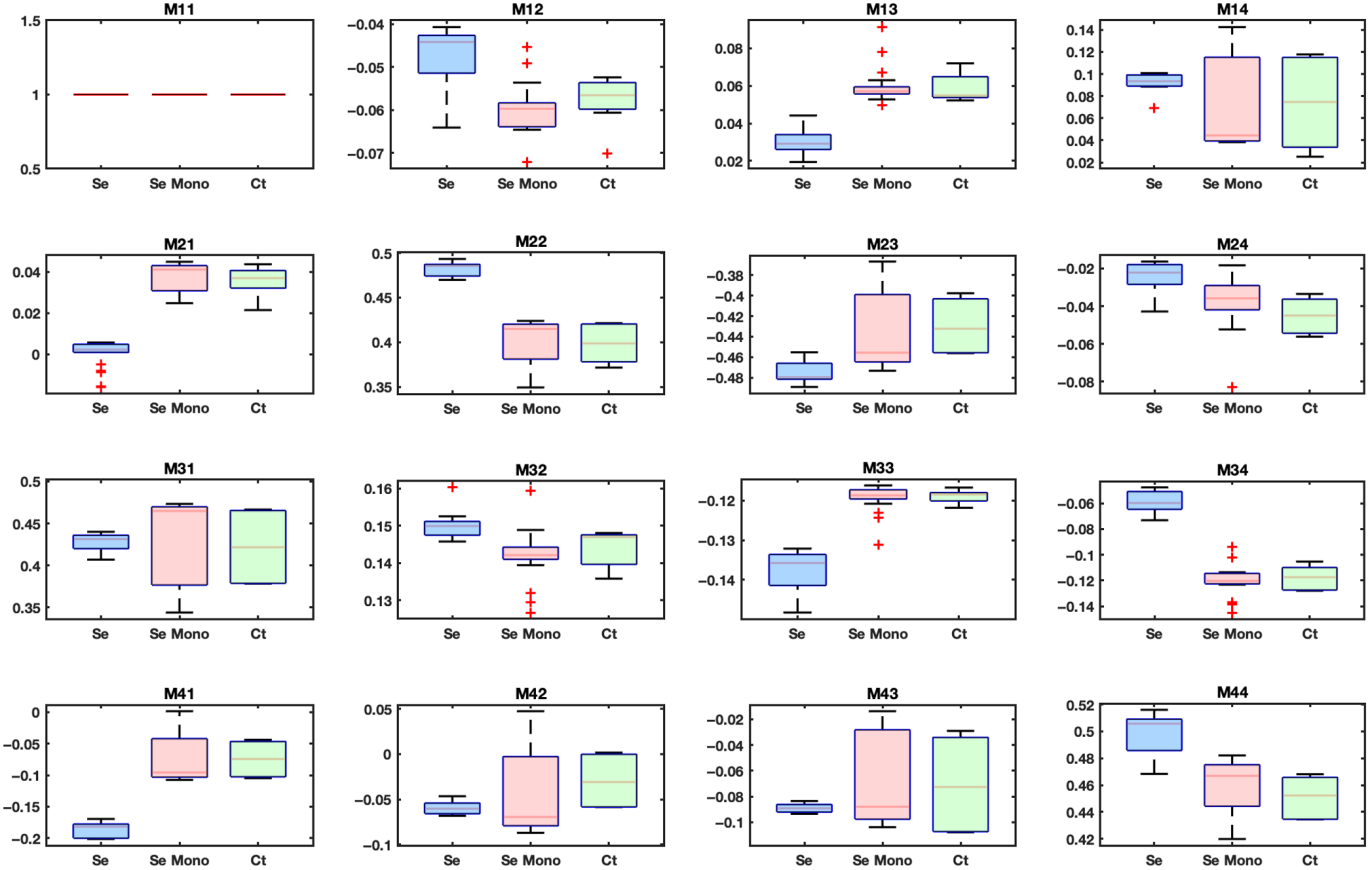
MM values of *Staphylococcus epidermidis* (*Se*), normal grown biofilm, the monolayer, and the control sample. We observed that grown biofilms have a smaller spread of data and different values compared with the control sample and the monolayer. The measurements for box plots are done on three sample discs per species, with four to five measurements per disc.

**Fig. 7:**
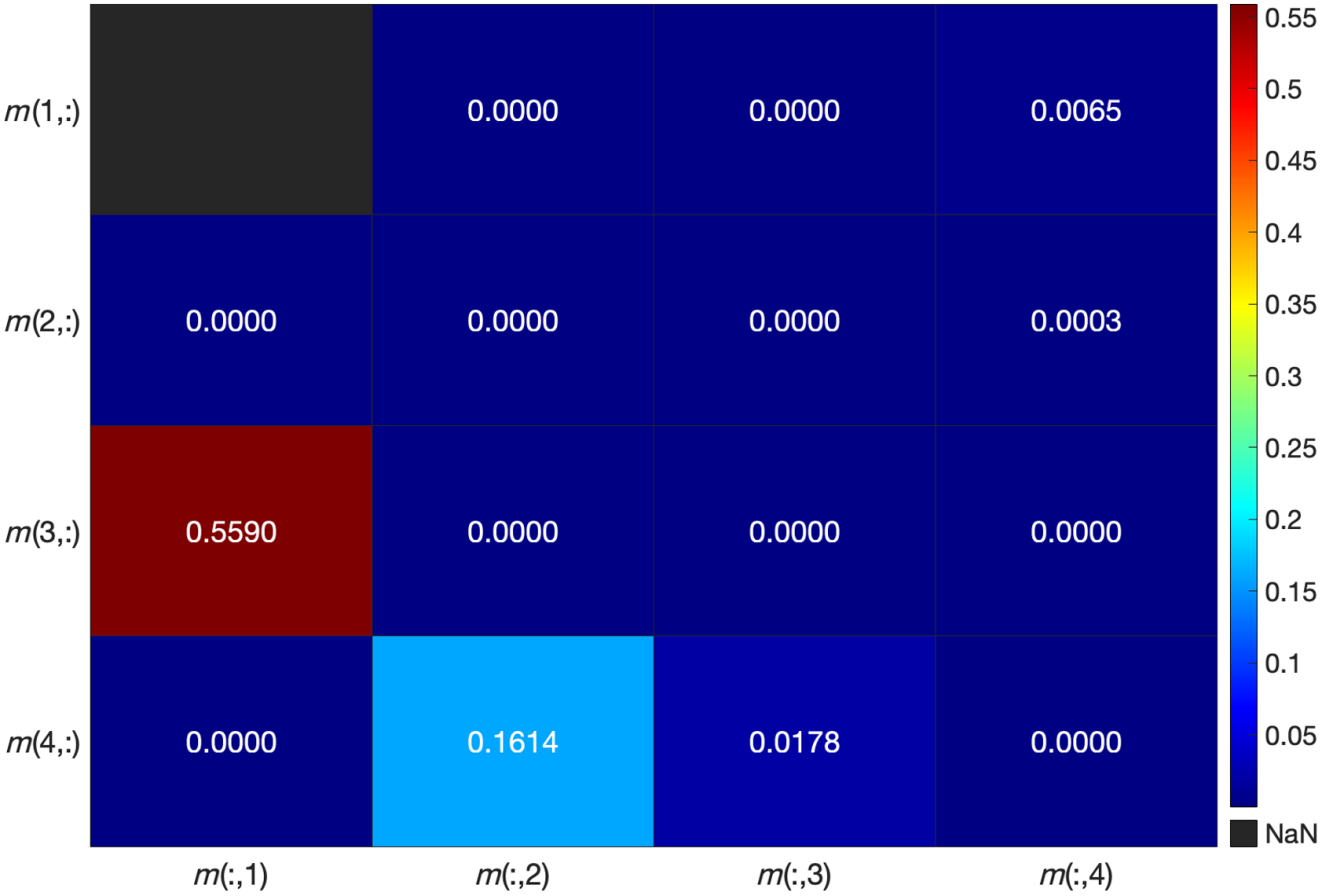
Heat map displaying p-values for the comparison between normally grown biofilms and the monolayer of *Staphylococcus epidermidis* (*Se*). The p-values indicate statistically significant differences between the two sample types. The blue color denotes a significant difference between the samples.

Bacterial biofilms are dynamic, anisotropic media with continuously varying properties. The thickness, structure, and growth of the biofilms determine their optical response. The optical behavior of a bacterial monolayer can be better understood in comparison to fully grown biofilms. In this study, we present an example case of *Staphylococcus epidermidis* and its monolayer. Figure 6 displays the boxplot of MM values for the bacterial species and the control sample. It is evident that the monolayer signals for almost all elements are closer to those of the control sample. There is a clear distinction between the control and sample values when compared with the fully grown biofilm. In particular, for the diagonal elements (m22, m33, and m44), the normal biofilm values deviate significantly from both the monolayer and control values. This trend is consistent for most other elements as well. To further quantify the optical behavior of the monolayer, we performed an ANOVA test followed by Tukey’s *post hoc* test. The results, presented as a heat map in Fig. 7, reveal statistically significant differences (p<0.05) between the monolayer and the fully grown biofilm for most elements. A similar conclusion could be drawn when all monolayers of the species were compared with normally grown biofilms. Taking the confocal images of the monolayer into account, we can attribute this difference to the fact that monolayers were most probably too thin to significantly influence the polarization response. The rationale behind including the monolayer condition was to establish a biologically meaningful lower bound for the presence of bacteria on a surface, without the structure of a grown biofilm. Although the monolayer did not induce significant changes in the Mueller matrix response, this finding is still informative as it shows that sparse bacterial adhesion is not enough to change the polarization state of the reflected light. By contrast, significant changes are exhibited by mature biofilms, confirming that the observed polarimetric response arises specifically from the three-dimensional structure and organization of bacterial colonies. These findings reinforce our confidence in the reliability of our approach and its potential for analyzing biofilm’s optical properties.

## 4 Conclusion

We successfully distinguished the five oral bacterial biofilms using Mueller matrix polarimetry, supported by robust statistical analysis, including ANOVA and *post hoc* tests, to ensure analytical rigor. The decomposition parameters polarizance, depolarization, and diattenuation proved effective in distinguishing the optical characteristics of the biofilms. Although the monolayer signals were analyzed, their indistinct results suggest that the layers are too thin to significantly influence the polarization response. As this is an initial study, the presented approach could be further developed to account for real clinical situations, such as the inclusion of multiple strains or co-cultures with human cells, making it more relevant for clinical applications. In particular, the concept could benefit from the combination with artificial intelligence for fast and essentially real-time analysis.

## Supplementary Material

10.1117/1.JBO.30.8.085001.s01

## Data Availability

The data associated with this paper is not public but can be made available upon reasonable request.

## References

[1] de Campos Kajimoto N. et al., “The oral microbiome of peri-implant health and disease: a narrative review,” Dent. J. 12(10), 299 (2024). DMJOD5 10.3390/dj12100299 PMC11506630 39452426

[2] Doornewaard R. et al., “How do peri-implant biologic parameters correspond with implant survival and peri-implantitis? A critical review,” Clin. Oral Implants Res. 29, 100–123 (2018). 10.1111/clr.13264 PMC6220966 30306697

[3] EPRD Deutsche Endoprothesenregister GmbH, Endoprothesenregister Deutschland Jahresbericht 2020, EPRD Deutsche Endoprothesenregister GmbH, Berlin, Deutschland (2020).

[4] Hashimoto Y. et al., “Microbial differences between active and remission peri-implantitis,” Sci. Rep. 12, 5284 (2022). SRCEC3 2045-2322 10.1038/s41598-022-09192-y 35347182 PMC8960758

[5] Anitua E. et al., “Assessing peri-implant bacterial community structure: the effect of microbiome sample collection method,” BMC Oral Health 24, 1001 (2024). 10.1186/s12903-024-04675-y 39187802 PMC11348724

[6] Carvalho E. B. et al., “Microbiota associated with peri-implantitis—a systematic review with meta-analyses,” Clin. Oral Implants Res. 34(11), 1176–1187 (2023). 10.1111/clr.14153 37523470

[7] Deutsche Gesellschaft für Implantologie im Zahn-, Mund- und Kieferbereich e.V., “Presseinformation 30.11.2018,” Online, 2018, https://www.dginet.de/documents/10164/1526375/PM_SCHWARZ.pdf/daa26dd8-0fe1-40b8-8969-f8e796623674 (Zugriff am 2025-01-09).

[8] Kommerein N. et al., “Development and characterization of an oral multispecies biofilm implant flow chamber model,” PLoS One 13(5), e0196967 (2018). POLNCL 1932-6203 10.1371/journal.pone.0196967 29771975 PMC5957423

[9] Chouirfa H. et al., “Review of titanium surface modification techniques and coatings for antibacterial applications,” Acta Biomater. 83, 37–54 (2019). 10.1016/j.actbio.2018.10.036 30541702

[10] Lee C.-T. et al., “Prevalences of peri-implantitis and peri-implant mucositis: systematic review and meta-analysis,” J. Dent. 62, 1–12 (2017). 10.1016/j.jdent.2017.04.011 28478213

[11] Arciola C. R. et al., “Biofilm formation in staphylococcus implant infections: a review of molecular mechanisms and implications for biofilm-resistant materials,” Biomaterials 33(26), 5967–5982 (2012). BIMADU 0142-9612 10.1016/j.biomaterials.2012.05.031 22695065

[12] Colombo A. Tanner A., “The role of bacterial biofilms in dental caries and periodontal and peri-implant diseases: a historical perspective,” J. Dent. Res. 98(4), 373–385 (2019). JDREAF 0022-0345 10.1177/0022034519830686 30890060

[13] Doll-Nikutta K. et al., “Bacteria-epithelial cell interaction influences cytotoxicity and antibacterial effect of silver-gold alloy nanoparticles on a species-specific level,” ChemNanoMat 10(2), e202300400 (2024). 10.1002/cnma.202300400

[14] Doll-Nikutta K. et al., “Adhesion forces of oral bacteria to titanium and the correlation with biophysical cellular characteristics,” Bioengineering 9(10), 567 (2022). BENGEQ 0178-2029 10.3390/bioengineering9100567 36290534 PMC9598062

[15] Parthasarathy R., “Monitoring microbial communities using light sheet fluorescence microscopy,” Biomed. Opt. Express 43, 31–37 (2018). BOEICL 2156-7085 10.1016/j.mib.2017.11.008 PMC5963963 29175679

[r16] Wolf G. Crespo J. Reis M., “Optical and spectroscopic methods for biofilm examination and monitoring,” Rev. Environ. Sci. Biotechnol. 1, 227–251 (2002). 10.1023/A:1021238630092

[17] Nguyen C. T. et al., “Noninvasive in vivo optical detection of biofilm in the human middle ear,” Proc. Natl. Acad. Sci. U. S. A. 109(24), 9529–9534 (2012). 10.1073/pnas.1201592109 22645342 PMC3386096

[18] Debener N. et al., “Optically accessible, 3D-printed flow chamber with integrated sensors for the monitoring of oral multispecies biofilm growth in vitro,” Front. Bioeng. Biotechnol. 12, 1483200 (2024). 10.3389/fbioe.2024.1483200 39588362 PMC11586212

[19] Larimer C. et al., “A method for rapid quantitative assessment of biofilms with biomolecular staining and image analysis,” Anal. Bioanal. Chem. 408, 999–1008 (2016). ABCNBP 1618-2642 10.1007/s00216-015-9195-z 26643074 PMC4709385

[20] Wenck C. et al., “Colorimetric detection of oral bacteria using functionalized gold nanoparticles as a plasmonic biosensor array,” Nanoscale Adv. 6(5), 1447–1459 (2024). 10.1039/D3NA00477E 38419865 PMC10898432

[21] Ho C.-S. et al., “Rapid identification of pathogenic bacteria using Raman spectroscopy and deep learning,” Nat. Commun. 10(1), 1–8 (2019). NCAOBW 2041-1723 10.1038/s41467-019-12898-9 31666527 PMC6960993

[22] Fricke D. et al., “Mueller matrix analysis of collagen and gelatin containing samples towards more objective skin tissue diagnostics,” Polymers 12(6), 1400 (2020). 10.3390/polym12061400 32580462 PMC7361993

[r23] Fricke D. Maas S. Wollweber M., “Liquid crystal retarders for fully automated fast measurement of the Mueller matrix of the skin without moving parts,” in Proc. 119th Annu. Meet. Ger. Soc. Appl. Opt. DGAO, Aalen, Germany (2018).

[24] Fricke D. et al., “Non-contact fast Mueller matrix measurement system for investigation of inflammatory skin diseases,” Proc. SPIE 10863, 1086307 (2019). PSISDG 0277-786X 10.1117/12.2509766

[25] Tuchin V. V., “Polarized light interaction with tissues,” J. Biomed. Opt. 21(7), 071114 (2016). JBOPFO 1083-3668 10.1117/1.JBO.21.7.071114 27121763

[r26] Novikova T. et al., “Special section guest editorial: polarized light for biomedical applications,” J. Biomed. Opt. 21(7), 071001 (2016). JBOPFO 1083-3668 10.1117/1.JBO.21.7.071001 27411079

[r27] Ghosh N. Vitkin I. A., “Tissue polarimetry: concepts, challenges, applications, and outlook,” J. Biomed. Opt. 16(11), 110801 (2011). JBOPFO 1083-3668 10.1117/1.3652896 22112102

[28] Jütte L. Roth B., “Mueller matrix microscopy for in vivo scar tissue diagnostics and treatment evaluation,” Sensors 22(23), 9349 (2022). SNSRES 0746-9462 10.3390/s22239349 36502051 PMC9740816

[29] Qi J. He H. Lin J., “Assessment of tissue polarimetric properties using stokes polarimetric imaging with circularly polarized illumination,” J. Biophotonics 11(4), e201700139 (2018). 10.1002/jbio.201700139 29131523

[30] Sharma G. et al., “Mueller matrix polarimetry for mouse brain tissue analysis,” Proc. SPIE 13322, 133220C (2025). PSISDG 0277-786X 10.1117/12.3042290

[31] Lee H. R. et al., “Digital histology of tissue with Mueller microscopy and FastDBSCAN,” Appl. Opt. 61(32), 9616–9624 (2022). APOPAI 0003-6935 10.1364/AO.473095 36606902

[32] He H. et al., “Mueller matrix polarimetry—an emerging new tool for characterizing the microstructural feature of complex biological specimen,” J. Lightwave Technol. 37(11), 2534–2548 (2019). JLTEDG 0733-8724 10.1109/JLT.2018.2868845

[33] Badieyan S. et al., “Detection and discrimination of bacterial colonies with Mueller matrix imaging,” Sci. Rep. 8(1), 10815 (2018). SRCEC3 2045-2322 10.1038/s41598-018-29059-5 30018335 PMC6050273

[34] Banerjee P. Soni J. Purwar H., “Probing the fractal pattern and organization of Bacillus thuringiensis bacteria colonies growing under different conditions using quantitative spectral light scattering polarimetry,” J. Biomed. Opt. 18(3), 035003 (2013). JBOPFO 1083-3668 10.1117/1.JBO.18.3.035003 23462968

[35] Manhas S. et al., “Demonstration of full 4 × 4 Mueller polarimetry through an optical fiber for endoscopic applications,” Opt. Express 23(3), 3047–3054 (2015). OPEXFF 1094-4087 10.1364/OE.23.003047 25836165

[36] Qi J. Elson D. S., “A high definition Mueller polarimetric endoscope for tissue characterisation,” Sci. Rep. 6, 25953 (2016). SRCEC3 2045-2322 10.1038/srep25953 27173145 PMC4865982

[37] Ushenko Y. A. et al., “Mueller-matrix of laser-induced autofluorescence of polycrystalline films of dried peritoneal fluid in diagnostics of endometriosis,” J. Biomed. Opt. 21(7), 071116 (2016). JBOPFO 1083-3668 10.1117/1.JBO.21.7.071116 27192944

[38] Dubolazov A. V. et al., “Birefringence images of polycrystalline films of human urine in early diagnostics of kidney pathology,” Appl. Opt. 55, B85–B90 (2016). APOPAI 0003-6935 10.1364/AO.55.000B85 27140137

[39] Sharma G. et al., “Enhanced oral bacterial discrimination by using Mueller matrix polarimetry,” Proc. SPIE 13322, 1332205 (2025). PSISDG 0277-786X 10.1117/12.3041861

[40] Flemming H.-C. Wingender J., “The biofilm matrix,” Nat. Rev. Microbiol. 8(9), 623–633 (2010).1740-1526 10.1038/nrmicro2415 20676145

[41] Foster T., “Staphylococcus,” in Medical Microbiology, 4th ed., University of Texas Medical Branch, Galveston, Texas (1996). 21413338

[42] Jutte L. et al., “Mueller matrix-based approach for the ex vivo detection of riboflavin-treated transparent biotissue,” Appl. Sci. 11(23), 11515 (2021). 10.3390/app112311515

[43] Sharma G. et al., “Monitoring of multiple fabrication parameters of electrospun polymer fibers using Mueller matrix analysis,” J. Opt. 26, 045404 (2024). 10.1088/2040-8986/ad2ca4

[44] Azzam R. M. A., “Stokes-vector and Mueller-matrix polarimetry,” J. Opt. Soc. Am. A 33(7), 1396–1408 (2016). JOAOD6 0740-3232 10.1364/JOSAA.33.001396 27409699

[45] Lu S.-Y. Chipman R. A., “Interpretation of Mueller matrices based on polar decomposition,” J. Opt. Soc. Am. A 13(5), 1106 (1996). JOAOD6 0740-3232 10.1364/JOSAA.13.001106

[46] Flemming H.-C. et al., “Biofilms: an emergent form of bacterial life,” Nat. Rev. Microbiol. 14(9), 563–575 (2016).1740-1526 10.1038/nrmicro.2016.94 27510863

[47] Rath H. Stumpp S. N. Stiesch M., “Development of a flow chamber system for the reproducible in vitro analysis of biofilm formation on implant materials,” PLoS One 12(2), e0172095 (2017). POLNCL 1932-6203 10.1371/journal.pone.0172095 28187188 PMC5302373

[48] Schmitt J. M. Kumar G., “Turbulent nature of refractive-index variations in biological tissue,” Opt. Lett. 21, 1310–1312 (1996). OPLEDP 0146-9592 10.1364/OL.21.001310 19876335

[49] Singh M. D. Vitkin I. A., “Discriminating turbid media by scatterer size and scattering coefficient using backscattered linearly and circularly polarized light,” Biomed. Opt. Express 12(11), 6831–6843 (2021). BOEICL 2156-7085 10.1364/BOE.438631 34858683 PMC8606157

[50] Cameron B. D. Li Y. Nezhuvingal A., “Determination of optical scattering properties in turbid media using Mueller matrix imaging,” J. Biomed. Opt. 11(5), 054031 (2006). JBOPFO 1083-3668 10.1117/1.2363347 17092180

[51] Kolenbrander P. E., “Multispecies communities: interspecies interactions influence growth on saliva as sole nutritional source,” Int. J. Oral Sci. 3(2), 49–54 (2011). 10.4248/IJOS11025 21485308 PMC3469885

[52] Buttner H. Mack D. Rohde H., “Structural basis of staphylococcus epidermidis biofilm formation: mechanisms and molecular interactions,” Front. Cell. Infect. Microbiol. 5, 14 (2015). 10.3389/fcimb.2015.00014 25741476 PMC4330918

[53] Hammerschmidt S. Rohde M. Preissner K. T., “Extracellular matrix interactions with gram-positive pathogens,” Microbiol. Spectr. 7(2), 10–1128 (2019). 10.1128/microbiolspec.GPP3-0041-2018 PMC11590433 31004421

[54] Okamura H. et al., “Outer membrane vesicles of Porphyromonas gingivalis: novel communication tool and strategy,” Jpn. Dent. Sci. Rev. 57, 138–146 (2021). 10.1016/j.jdsr.2021.07.003 34484474 PMC8399048

[55] Chapuis C. Rosso L. Flandrois J., “Relationship between colonial surface and density on agar plate,” J. Appl. Bacteriol. 79(5), 542–550 (1995). JASUEH 10.1111/j.1365-2672.1995.tb03175.x

[56] Alali S. Vitkin A., “Polarized light imaging in biomedicine: emerging Mueller matrix methodologies for bulk tissue assessment,” J. Biomed. Opt. 20(6), 061104 (2015). JBOPFO 1083-3668 10.1117/1.JBO.20.6.061104 25793658

[57] Li P. et al., “Polaromics: deriving polarization parameters from a Mueller matrix for quantitative characterization of biomedical specimen,” J. Phys. D Appl. Phys. 55, 034002 (2022). 10.1088/1361-6463/ac292f

[58] Azzam R. M. A., “Propagation of partially polarized light through anisotropic media with or without depolarization: a differential 4 × 4 matrix calculus,” J. Opt. Soc. Am. 68, 1756–1767 (1978). JOSAAH 0030-3941 10.1364/JOSA.68.001756

[59] Gompf B. et al., “On the depolarization in granular thin films: a Mueller-matrix approach,” J. Opt. Soc. Am. A 35(2), 301–308 (2018). JOAOD6 0740-3232 10.1364/JOSAA.35.000301 29400879

[60] He C. He H. Li X., “Quantitatively differentiating microstructures of tissues by frequency distributions of Mueller matrix images,” J. Biomed. Opt. 20, 105009 (2015). JBOPFO 1083-3668 10.1117/1.JBO.20.10.105009 26502227

