# Supplementary material for: Label-free distinction of implant infection–associated bacterial biofilms by Mueller matrix polarimetry
Source: J Biomed Opt. 2025 Aug 22;30(8):085001. doi: 10.1117/1.JBO.30.8.085001 (PMC12371480; doi:10.1117/1.JBO.30.8.085001)
Supplement: Supplementary file 1 [file JBO_030_085001_SD001.pdf]

Supplementary material: Label-free distinction of implant infection-associated bacterial biofilms by Mueller matrix polarimetry

Table 1 F-values for all MM elements

	M[:,1]	M[:,2]	M[:,3]	M[:,4]
**M[1,:]**	NaN	14.7239	55.1755	8.9731
**M[2,:]**	66.8061	207.6625	19.8960	139.4428
**M[3,:]**	0.7280	53.7153	66.8581	171.6871
**M[4,:]**	183.5239	30.4935	25.0498	43.7802

Table 2 Post-hoc results on bacterial biofilms showing the significant mean differences of MM elements (p<0.05). 'n.s.' stands for not significant difference between the species.

**M11**
No significant differences detected

**M12**						
**Species**	**Sa**	**Se**	**Pg**	**Sm**	**So**	**Ct**
**Sa**	0	-0.0148	-0.0146	-0.0204	-0.014	n.s.
**Se**		0	n.s.	n.s.	n.s.	0.004
**Pg**			0	n.s.	n.s.	0.0054

**Sm**				0	0.0002	0.0099
**So**					0	0.0032
**Ct**						0

M13

**Species**	**Sa**	**Se**	**Pg**	**Sm**	**So**	**Ct**
**Sa**	0	n.s.	0.0061	0.0089	0.0031	0.0285
**Se**		0	n.s.	0.0023	n.s.	0.0352
**Pg**			0	n.s.	n.s.	0.0399
**Sm**				0	n.s.	-0.044
**So**					0	0.0385
**Ct**						0

M14

**Species**	**Sa**	**Se**	**Pg**	**Sm**	**So**	**Ct**
**Sa**	0	n.s.	-0.0342	-0.0381	n.s.	n.s.
**Se**		0	-0.0332	-0.0371	n.s.	n.s.
**Pg**			0	n.s.	n.s.	0.0155
**Sm**				0	0.0014	0.0159
**So**					0	0.011
**Ct**						0

M21

**Species**	**Sa**	**Se**	**Pg**	**Sm**	**So**	**Ct**
**Sa**	0	n.s.	0.0004	n.s.	n.s.	0.0419
**Se**		0	n.s.	n.s.	n.s.	0.0429
**Pg**			0	n.s.	n.s.	0.0476

**Sm**				0	n.s.	0.0443
**So**					0	0.0458
**Ct**						0

M22

**Species**	**Sa**	**Se**	**Pg**	**Sm**	**So**	**Ct**
**Sa**	0		-0.0475	-0.0516	-0.0466	0.0648
**Se**		0	-0.0444	-0.0485	-0.0435	0.0679
**Pg**	0.0475	0.0444	0			0.1031
**Sm**	0.0516	0.0485		0		0.105
**So**	0.0466	0.0435			0	0.0995
**Ct**	-0.0648	-0.0679	-0.1031	-0.105	-0.0995	0

M23

**Species**	**Sa**	**Se**	**Pg**	**Sm**	**So**	**Ct**
**Sa**	0	n.s.	n.s.	n.s.	n.s.	0.0599
**Se**		0	n.s.	n.s.	n.s.	0.0583
**Pg**			0	n.s.	n.s.	0.0559
**Sm**					n.s.	0.0571
**So**		-			0	0.0553
**Ct**						**0**

M24

**Species**	**Sa**	**Se**	**Pg**	**Sm**	**So**	**Ct**
**Sa**	0	n.s.	0.037	0.0354	0.0355	0.0174
**Se**		0	0.0319	0.0303	0.0304	0.0123
**Pg**			0	n.s.	n.s.	0.0253

**Sm**					0	0.0251
**So**					0	0.0255
**Ct**						0

M31

No significant No significant differences detected						

M32

**Species**	**Sa**	**Se**	**Pg**	**Sm**	**So**	**Ct**
**Sa**	0	-0.0111	-0.0207	-0.0265	-0.0218	n.s.
**Se**		0	-0.0148	-0.0206	-0.016	0.0004
**Pg**			0	-0.0098	-0.016	0.0111
**Sm**				0	n.s.	0.016
**So**					0	0.0111
**Ct**						0

M33

**Species**	**Sa**	**Se**	**Pg**	**Sm**	**So**	**Ct**
**Sa**	0	0.0013	0.0039	0.0122	0.0043	0.0186
**Se**		0	0.0036	0.006	n.s.	0.0247
**Pg**			0	0.0047	n.s.	0.0262
**Sm**				0	-0.0125	0.0354
**So**					0	0.0277
**Ct**						0

M34

**Species**	**Sa**	**Se**	**Pg**	**Sm**	**So**	**Ct**
**Sa**	0	n.s.	-0.0146	-0.0283	-0.019	0.0493
**Se**		0		-0.0263	-0.0171	0.0513
**Pg**			0	-0.0197	n.s.	0.0578
**Sm**				0	0.0017	0.07
**So**					0	0.0604
**Ct**						0

M41

**Species**	Sa	Se	Pg	Sm	So	Ct
**Sa**	0	0.0002	0.0289	0.0417	0.0275	0.1117
**Se**		0	0.0131	0.026	0.0117	0.1274
**Pg**			0	0.0011	n.s.	0.1525
**Sm**				0	n.s.	0.1684
**So**					0	0.1547
**Ct**						0

M42

**Species**	**Sa**	**Se**	**Pg**	**Sm**	**So**	**Ct**
**Sa**	0	n.s.	-0.0534	-0.0526	-0.0526	0.0486
**Se**		0	-0.0495	-0.0487	-0.0488	0.0447
**Pg**			0	n.s.	n.s.	n.s.
**Sm**				0	n.s.	n.s.
**So**					0	0.017
**Ct**						0

M43

**Species**	**Sa**	**Se**	**Pg**	**Sm**	**So**	**Ct**
**Sa**	0	n.s.	0.0155	0.0127	0.0116	n.s.
**Se**		0	0.0155	0.0126	0.0116	n.s.
**Pg**			0	n.s.	n.s.	0.0641
**Sm**				0	n.s.	0.0644
**So**					0	-0.064
**Ct**						0

M44

**Species**	Sa	Se	Pg	Sm	So	Ct
**Sa**	0	n.s.	-0.0302	-0.0355	-0.0299	0.0272
**Se**		0	-0.0286	-0.0339	-0.0283	0.0289
**Pg**			0	n.s.	n.s.	0.0469
**Sm**				0	n.s.	0.0496
**So**					0	0.0434
**Ct**						0
